# Benzodiazepine Use and Misuse Among Patients in a Methadone Program

**DOI:** 10.1186/1471-244X-11-90

**Published:** 2011-05-19

**Authors:** Kevin W Chen, Christine C Berger, Darlene P Forde, Christopher D'Adamo, Eric Weintraub, Devang Gandhi

**Affiliations:** 1 Center for Integrative Medicine University of Maryland School of Medicine 520 W. Lombard St., East Hall.Baltimore, MD 21201, USA; 2 Department of Psychiatry University of Maryland School of Medicine 701 W. Pratt Street Baltimore, MD 21201, USA

**Keywords:** Benzodiazepines use, prescription drug misuse, methadone program, anxiety, survey study

## Abstract

**Background:**

Benzodiazepines (BZD) misuse is a serious public health problem, especially among opiate-dependent patients with anxiety enrolled in methadone program because it puts patients at higher risk of life-threatening multiple drug overdoses. Both elevated anxiety and BZD misuse increase the risk for ex-addicts to relapse. However, there is no recent study to assess how serious the problem is and what factors are associated with BZD misuse. This study estimates the prevalence of BZD misuse in a methadone program, and provides information on the characteristics of BZD users compared to non-users.

**Methods:**

An anonymous survey was carried out at a methadone program in Baltimore, MD, and all patients were invited to participate through group meetings and fliers around the clinic on a voluntary basis. Of the 205 returned questionnaires, 194 were complete and entered into final data analysis. Those who completed the questionnaire were offered a $5 gift card as an appreciation.

**Results:**

47% of the respondents had a history of BZD use, and 39.8% used BZD without a prescription. Half of the BZD users (54%) started using BZD after entering the methadone program, and 61% of previous BZD users reported increased or resumed use after entering methadone program. Compared to the non-users, BZD users were more likely to be White, have prescribed medication for mental problems, have preexistent anxiety problems before opiate use, and had anxiety problems before entering methadone program. They reported more mental health problems in the past month, and had higher scores in anxiety state, depression and perceived stress (p < .05).

**Conclusions:**

Important information on epidemiology of BZD misuse among methadone-maintenance patients suggests that most methadone programs do not address co-occurring anxiety problems, and methadone treatment may trigger onset or worsening of BZD misuse. Further study is needed to explore how to curb misuse and abuse of BZD in the addiction population, and provide effective treatments targeting simultaneously addiction symptoms, anxiety disorders and BZD misuse.

## Background

Benzodiazepines (BZD) misuse and abuse is a serious public health problem in the United States. This problem is especially pertinent among those with opiate dependence [1] because these individuals are more likely to experience elevated anxiety after stopping use of opiates, with increased risk of using BZD as an anxiety coping strategy [2]. In addition, it has been shown that individuals who abuse BZD are at increased risk of continuing opiate abuse [3]and failing to stay in methadone treatment [4,5]. BZD use has also been shown to be associated with use of multiple psychotropic drugs, higher rates of depression and anxiety [6].

Benzodiazepines are psychoactive drugs used primarily to treat anxiety and sleep disorders. Their intended uses include anxiolytic, sedative hypnotic, anticonvulsant, and muscle relaxant [7] therapy in low to medium doses. They are central nervous system depressants and research has shown that inappropriate use can result in physical [8] and psychological dependence [9] and increased personal harm and criminal activity [10]. BZD were initially developed and prescribed in small doses. Over time they have come to be prescribed in larger doses [7] which resulted in an increase in prescription abuse and/or use without a prescription. These two problems have created a public health issue identified as benzodiazepine misuse [7,11-14]

Benzodiazepine misuse and abuse are challenging to define. Criteria used to define BZD dependence have included: unsuccessful attempts to cut back or terminate use, feeling uncomfortable when not taking BZD [9], history of long-term use, and dosage escalation and high anxiety levels despite taking BZD [1]. There are three 3 sub-populations who misuse BZD according to Ashton [1]: (1) *patients who are prescribed BZD therapeutically for the short-term and take them for the long-term*, estimated at 4 million people in the U.S. and it is likely that half of them are dependent; (2) *patients who are prescribed BZD therapeutically but then increase the dose on their own by going to additional doctors or seeking them out on the street *(prevalence unknown), and (3*) patients who seek BZD for recreational use without a prescription*, which might represent a small proportion of BZD abusers though at present there is no estimate on the actual prevalence. This third group tends to be poly-substance abusers and they seek BZD to enhance the effects of other drugs, alleviate withdrawal effects of other drugs, and to produce their own stimulating effects taken alone or intravenously. For those who take BZD for anxiety, the effects were reported to wear off more quickly over time and individuals were more likely to try higher doses or add other BZD to minimize discomfort. It thus appears that continued BZD use does not produce a reduction of anxiety, but rather, keeps withdrawal symptoms at bay [1]

Busto et al. [15] found that BZD were the primary drug of abuse in 32% of the multiple drug abusers. Anecdotal reports from our clinical observations and counselor feedback seem to indicate that a large proportion of patients on methadone maintenance use BZD without a prescription (misuse). Lankenau et al. [16] noted increases in prescription drug misuse including BZD among patients of a methadone program. Their findings suggested that BZD misuse might be associated with increased use of other illegal drugs and drug dependency and suggested increased street outreach as a method to address this problem. However, due to lack of education regarding monitoring techniques for physicians and inaccurate reporting [17] from patients, a comprehensive grasp of this problem has been elusive. It seems that anxiety could be the primary motivation for this misuse but there are few studies at this time to develop a comprehensive picture of this social and health problem. A PUBMED search using keywords "benzodiazepine abuse" and "opiate" revealed only seven studies conducted in the past twenty years [3,5,6,18-21]. Therefore, it seems that there is a discrepancy between clinical reports of BZD misuse and research investigation of the problem, particularly in the United States.

There has been little recent research into BZD use among methadone maintenance patients in the United States. Small studies were conducted during the 1980s and 1990s but need updating due to the changing demographics and patterns of drug use. Gelkopf et al. [22] examined BZD abuse in methadone maintenance patients in a one-year prospective study in an Israeli clinic and found that lifetime prevalence of BZD abuse was 66.3% and current prevalence of BZD abuse was 50.8%. This study indicates that BZD abuse seems to be a problem for heroin addicts both before entering and during their methadone treatment.

This study is among the few in the U.S. [23] that closely examines BZD use and misuse among methadone-maintained patients and was conducted to provide more current data. These data will provide the background information necessary to develop more acceptable and effective therapies for the treatment of BZD misuse in opiate dependent patients in the future.

The main purposes of this survey study are: 1) to estimate the prevalence of BZD use and misuse among patients in a methadone program; 2) to determine the main reasons for their BZD use or misuse, to evaluate whether the methadone treatment was a trigger for new, increased or resumed use of BZD; 3) to examine the characteristics of BZD users that may differentiate them from other opiate-dependent patients, and 4) to assess what proportion of BZD users are willing to accept treatment if it were available.

## Methods

### Study Setting and Subjects

The survey was conducted at a methadone treatment program in Baltimore city. This program is a part of the University of Maryland Medical Center. The program provides methadone maintenance services to about 500 insured and uninsured (mainly grant-funded) patients who were the targeted population for this anonymous survey.

All patients enrolled in the treatment program between December 2009 and July 2010 were eligible to participate in the study. Participation was voluntary, and the instructions on the first page of the survey instrument asked participants not to write their name anywhere on the questionnaire. Each participant who completed the questionnaire was offered a $5 gift card as an appreciation for their participation. Measures were taken to ensure that the same participant did not fill out the survey twice.

### Procedure

The most effective way to reach all patients in such a clinic may be to let the counselors give the questionnaire to each client in their caseload. However, since patients who used BZD were often given negative consequences (such as increased counseling or removal of take-home privileges) we were concerned that patients might not answer the questionnaire truthfully if they thought, despite our assurance to the contrary, that their counselor might find out their response to the study questions. We therefore took special measures to minimize the program counselors' involvement in data collection. We mainly used two methods of recruiting patients into this survey: (1) we collaborated with the clinic counselors to attend their weekly group meetings and asked all patients in each group to participate in the 20-30 minute survey on a voluntary basis. The counselors left the room during study administration while our research staff supervised the questionnaire administration and answered questions. (2) For those patients who did not attend any group meetings for various reasons, we set up two one-hour walk-in study sessions a week in the clinic and posted fliers around the clinic to invite patients to participate in a study on their health status. We avoided listing BZD in the fliers so as to circumvent patient concerns about revealing their BZD use status. Meanwhile, the counselors were asked to send those patients who did not attend group meetings to these study sessions. Study participation was open to all patients regardless of any history of BZD use to prevent inadvertent identification of BZD users due to their volunteering to participate in the study. A research staff member was there to supervise the questionnaire administration and answer questions.

To make sure that patients understood what BZD are, we gave the following specific instructions on the first page: "Benzodiazepines, known as Benzos, are tranquilizer pills that are prescribed by doctors for treating stress, nerves, anxiety or sleeping problems. Other names may include Valium, Xanax, Librium, Ativan, Klonopin, nerve pills, etc. You may know them as pins, bars, or footballs. A list of names and pictures are available if you are not sure what Benzos are. You are invited to participate even if you have never used Benzos."

Since this was a minimum-risk anonymous survey, participation was completely voluntary, and no name or any other identifiers appeared on the study form, the study was approved by University of Maryland Baltimore institutional review board (IRB) for a waiver of informed consent.

### The Questionnaire

Based on some observations and meetings with counselors, we developed a 5-page questionnaire assessing basic demographics, substance use, and the health issues related to our study aims. The key questions related to BZD use included: what were the main reasons you first began to use opiates? Have you ever used any benzodiazepines? Was your initial BZD use a prescription from your doctor(s)? Have you ever used any BZD without a prescription? What were the reasons you started to use BZD that were not prescribed to you in the first place? Did you use any BZD before you entered the methadone program? Did your BZD use increase or start after coming to the methadone program? How many days did you use any BZD in the past 30 days? (The actual Questionnaire is available upon request)

In order to understand the possible differences in psychological profile between BZN users and non-users, we also included a few standard self-report psychological assessments like the Spielberger State Anxiety Inventory [24], Anxiety Sensitivity Index [25], CES depression scale [26] and Perceived Stress Scale [27]. We hypothesized that BZN users might have a different psychological profile from the non-users, which should be reflected in their mood, anxiety and perceived stress in life. We tried to minimize the number of psychological scales in such a quick survey to ensure reliability of the answers.

### Statistical Analysis

All data analyses were conducted using SPSS for Windows (version 18). The population was described using frequency analyses. Cross-tabulations for categorical variables, and ANOVA for continuous variables, were performed between each predictive variable according to BZD use status to examine the possible differences. Pearson's Chi square tests for categorical variables, and F tests in ANOVA for continuous variables, were calculated. Multivariable logistic regression modeling was performed to examine the significant predictors (correlates) of BZD use with control for other possible confounders.

## Results

### Basic demographics and substance use

We collected a total of 205 returned questionnaires; 194 of them were judged to meet a minimum completion threshold and were included in the final data analysis. To evaluate the possible selection bias of our sample, we compared the demographic characteristics of our sample (n = 194) with the total patient population at the methadone clinic (n = 485 by the end of 2010). Of the 194 participants in our sample, 43.3% were female (compared to 39.0% in the total patient population); 21.9% were white or Caucasian (25.9% in the total patient population), 75.9% Black or African American (69.9% in the total patient population). The demographic characteristics were not statistically significant between our sample and the total methadone program population, suggesting that there was very little evidence of selection bias in this survey study. In addition, 28.3% of survey participants were currently married or living with a partner, 47.6% never married; 79.1% had education level of high school diploma or less; 67.8% had at least one child (75% of them have multiple children); 12.5% of them hold a full-time job and another 12.5% had a part-time job, 35.9% were unemployed, and 28.3% had a disability income. Most participants considered themselves religious (90%); 35.5% of respondents considered their treatment prompted or suggested by criminal justice system or a court (20% were actually on parole or probation). Participants' age ranged from 17 to 83 years old, with a mean of 46.6 years, and median age at 47 (which was comparable to the median age of 48 in the total patient population).

Forty-three percent of respondents reported some form of chronic medical problem that continued to affect their life; 31% took some prescribed medications on a regular basis for a physical health problem, and 30% took medications for mental or emotional problems (including anxiety and depression). About 48% of respondents reported having some form of anxiety or sleeping problems before they started using opiates/heroin; and 61.5% felt that they had some form of anxiety or sleeping problems before entering the methadone program, which could be the basis for their subsequent BZD use.

### Benzodiazepines use and misuse

Of the 191 respondents who answered the question on BZD use, 90 (47%) reported using BZD with or without a prescription. Of the 90 respondents who ever used BZD, only 25% said that their initial use began with a prescription; 84% of them acknowledged ever using BZD without a prescription (misuse) (some of them started BZD use with a prescription, but used it later without a prescription). Therefore, of the total effective sample, 39.8% or 76 respondents reported ever using BZD without a prescription. The main reasons they gave for using BZD without a prescription are listed in Table 1. Curiosity was the most common reason (46%), followed by relieving tension or anxiety (41%) and feeling good (37%).

**Table 1:** List of the main reasons for starting misuse of BZD

The reasons for initial use of opiates	N	%
Curious to see what it's like	41	45.6

To relax or relieve tension/anxiety	37	41.1

To feel good	33	36.7

To get high	22	24.4

To overcome depression or frustration	21	23.3

To get away from my problem or troubles	18	20.0

To have a good time with my friends	13	14.4

To go along with what my friends are doing	9	10.0

It's something my friends do when we get together	7	7.8

To fit in with a group I like	6	6.7

To produce intense, exciting experience	6	6.7

To rebel against my parent(s)	3	3.3

Never used non-prescribed BZD	8	8.9

Among the BZD users, 54% did not start using BZD until after entering the methadone program. The mean age of onset into BZD use was around 31 years old. Of those who used BZD before entering the methadone program, 61% reported that their BZD use increased or restarted after entering the methadone program. Although 78% of BZD users did not acknowledge their BZD use as a problem at the moment, 56% of them had tried to stop using BZD at least once (28% of them tried to stop using BZD more than once, and 14% had entered in a BZD detoxification program). We asked the respondents if they would consider reducing or stopping use of BZD if we could provide help that will work; 40% said "Yes, definitely", 7% said "Maybe", and only 19% said "No" (33% had stopped using BZD already).

### Differences between BZD-users and non-users

Table 2 presents differences between BZD-users and non-users in this survey sample. Among those noticeable differences, BZD users were more likely to be of White or Caucasian race (35% vs. 11%, p < .01), have lower self-reported religiosity on the 1--10 scale (6.2 vs. 7.1, p < .01), and feel less healthy (35% vs. 48%; p < .05). Compared to non-users, BZD users were more likely to have been prescribed medication for mental or emotional problems (49% vs. 22%, p < .01), had anxiety problems before use of opiates (61% vs. 37%, p < .01), and had anxiety or sleep problems before entering the methadone program (78% vs. 48%, p < .01). They reported more days with mental or emotional problems in the past 30 days (10.4 vs. 6.3), and higher scores in all four psychological measures -- anxiety sensitivity, anxiety state, depression and perceived stress (p < .05).

**Table 2:** Comparison of Main Demographics and Health History Between BZD Users and Non-Users in the Methadone programs

Variables	Non-Users (n ≤ 101)	BZN-Users (n ≤ 90)	p ≤ ^a^
*Demographics*			

% female	40.4%	48.1%	.442

Mean age (SD)	47.5 (7.1)	45.4 (10.3)	.107

% of White/Caucasian	11.1%	35.3%	.001

% of Single (never married)	53.5%	42.7%	.369

Religiosity (1--10 scale)	7.12 (1.9)	6.17 (2.3)	.003

*Health Issues*			

General Health Status			
➤ Poor ➤ Ok, but below average ➤ Fair ➤ Good ➤ Excellent	7.1 11.1 33.3 45.5 3.0	1.1 26.1 37.5 31.8 3.4	.017

% had chronic medical problems	36.7%	47.5%	.153

% ever had prescribed med for mental Or emotional problems	21.6%	40.0%	.007

% have had anxiety or sleeping problems before starting use of opiates/heroin	36.8%	61.2%	.005

% have had anxiety or sleeping problems before entering methadone program	48.4%	78.3%	.001

Age of onset into alcohol use	16.5 (6.5)	14.9 (4.6)	.073

Age of onset into opiate/heroin use	23.5 (7.7)	21.7 (7.5)	.120

Age first admitted to a methadone program	37.1 (15.3)	35.3 (11.3)	.387

# of days with mental or emotional problems In the past 30 days	6.28 (9.9)	10.4 (11.6)	.048

*Psychological Measurements*			

Anxiety sensitivity - physical score	7.94 (6.1)	8.96 (5.4)	.231

Anxiety sensitivity - cognitive score	4.68 (5.4)	6.54 (5.4)	.020

Anxiety sensitivity - social score	5.78 (5.2)	6.98 (5.1)	.115

Anxiety sensitivity - Total score	18.4 (15.1)	22.4 (13.7)	.058

Perceived Stress Scale	15.6 (8.2)	18.6 (7.4)	.011

CES Depression scale	37.8 (10.4)	42.7 (8.5)	.001

Spielberg Anxiety State score	40.9 (11.6)	44.4 (10.6)	.036

a. P values are from chi-square test for categorical variables in contingency table, and F test continuous variables in ANOVA.

We further examined the differences in reasons given for initiation of opiate use, and discovered that the primary reason given by non-users was curiosity (59%), followed by social reasons (55%), whereas the number one reason for opiate use given by current BZD-users was "for pleasure or to get high" (67%), followed by social reason (63%). A significantly higher proportion of BZD users indicated both for pleasure or to get high (67% vs. 44%, p < .01) and to relieve negative mood (57% vs. 34%, p < .05) as reasons for opiate use compared to non-users. We also asked the respondents to check the noticed side effects after using opiates. As presented in Table 3, BZD users tended to report more negative effects of opiates than non-users by endorsing statements such as "feel tired and unhealthy", "do not want to go to work" and "cannot stay focused."

**Table 3:** Noticed Side Effects after Using Heroin/Opiates by BZD Use Status (Among those who answered the question N = 181)

Noticed Side Effects of Opiate Use	Non-Users (n = 93)	Past-Users (n = 41)	Current-Users (n = 47)	p ≤
Cost me too much money	66.7%	85.4%	66.0%	.064

Feel tired and unhealthy	25.8%	31.7%	46.8%	.043

Feel sleepy most of time	20.4	12.2	21.3	.467

Loss of interest in sex	31.2	31.7	40.4	.525

Do not want to go to work	21.5	24.4	42.6	.027

Loss of appetite	34.4	41.5	36.2	.736

Cannot stay focused	12.9	31.7	40.4	.001

Feel anxious or edge	22.6	36.6	40.4	.059

Not at all	14%	5%	10.6%	.300

To systematically examine the significant correlates of BZD use with control for possible confounders, we applied a logistic regression model with BZD-use status as the dependent variable and the possible significant correlates (see Table 2) as the independent variables (predictors). With the method of backward deletion in the multivariate logit model, four significant correlates that predict BZD usage status (see Table 4 for details) were revealed. They were White race, anxiety problem before entering methadone program, use of opiates for pleasure or to get high, and high depression score. The model explains 26% of variance (R^2^).

**Table 4:** Coefficients in Logistic Regression of Significant Predictors for BZD Use (Among those who had a valid responses to all related questions n = 184)

Predictors	Beta	s,e,	Odds ratio	p ≤
(Constant)	-3.357	0.805		.001

White (vs. Black and others)	1.001	0.423	2.72	.026

Had anxiety or sleeping problem before entering methadone program	0.867	0.341	2.38	.011

Used opiates for pleasure or get high	0.963	0.335	2.62	.004

CES Depression score	0.048	0.018	1.05	.007

Model Chi-square	39.27	(df = 5)		.001

Nagelkerke R^2^	0.257			

We noticed significant correlations among some predictors so that they could not be significant in the model simultaneously. For example, "use opiates for relieving tension or anxiety" was also a significant predictor (p < .05) if the predictor "use opiate for pleasure or to get high" was removed, which supports the assumption that BZD use could be the result of coping with stress and anxiety during methadone maintenance.

## Discussion

This cross-sectional survey study offered us answers to most of the research questions we asked for this study. First, we wanted to estimate the prevalence of BZD use and misuse among patients in a methadone program, and the survey revealed a prevalence of 47% lifetime use of BZD among our methadone-maintained patients, and most of whom used BZD without a prescription (39.8% of the survey respondents). This prevalence is lower than that reported in European countries [4,6]. This was likely due in part to the fact that our methadone maintenance program had a policy that no BZD use was allowed.

Second, we wanted to determine the main reasons for their BZD use or misuse, to evaluate whether the methadone treatment was a trigger for new, increased or resumed use of BZD; The survey shows that the main reasons for using BZD without a prescription are curiosity (46%), relieving tension or anxiety (41%) and feeling good (37%). Of all the self-reported BZD users, half (54%) did not use BZD until after they entered into methadone program, and 61% of previous BZD users reported increased use or resumption of use after entering the methadone program, which suggests a need for further research into reason for high prevalence of anxiety problems and BZD misuse in methadone-maintained patients.

Third, we wanted to examine the characteristics of BZD users that may differentiate them from other opiate-dependent patients, Our study revealed that, compared to those methadone-maintenance patients who never used BZD, BZD users were more likely to be White or Caucasian, have lower religiosity, have been prescribed medication for mental problems, have anxiety problems before entering the methadone program, and have preexistent anxiety problems before use of opiates. At the time of survey, they reported more number of days with mental or emotional problems in the past month, and higher scores in anxiety state, depression and perceived stress (p < .05).

Fourth, we wanted to assess what proportion of BZD users are willing to accept treatment if it were available. We did ask a question at the end of survey***, "****Do you intend to reduce or stop your use of Benzos if we can provide help that will work?" *Among those who are current BZD users, 60% answered "Yes, definitely", another 11% said they may try. Only 29% said they were not interested in stopping BZD use.

This is one of the few studies of its kind in the United States, and the first to provide data from a contemporary methadone maintenance population, especially on the possible characteristic differences between BZD-user and non-users, and on the possible impact of methadone treatment itself on the BZD use by opiate-dependent patients. There are several limitations in this study and caution is needed when interpreting the results and their implications. First, this is a cross-sectional survey, and causal relationships cannot be drawn from any of the data. Second, due to the specific research design in data collection, we might have missed two groups from the clinic. Namely, those with serious poly-drug use, mental health problems who might not attend any groups regularly and who may not want to reveal their problems in a study like this, and those who had take-home medication privileges of more than a week who would not need to come for frequent groups or to the clinic during the hours of our study sessions. These two groups represented two poles of this treatment population, and it is likely that they were under-represented in this survey. Third, it is not possible to determine any clinical diagnoses of co-occurring mental disorders through a survey like this; therefore, this study cannot establish a connection between BZD use and co-occurring mental disorders, even though it is possible that there may be such an association. It is also not clear from this survey if the apparent new onset of BZD use after starting methadone maintenance is intrinsically related to the treatment itself (e.g., medication adverse effects), or to some environmental factor such as increased association with other users and greater access to BZD.

## Conclusion

Despite these limitations, this survey study provides us with important information on the epidemiology of BZD use and misuse among methadone-maintenance patients. The study findings suggest that most methadone programs do not address co-occurring anxiety problems, and methadone treatment may trigger onset or worsening of BZD misuse.

Additionally, our findings shed light on the factors or correlates associated with BZD use by methadone maintenance patients. Further study is needed to explore ways to curb the use and abuse of prescription drugs like BZD in this population, and to develop effective treatments that will simultaneously target addiction symptoms, anxiety disorders, and BZD misuse.

## Competing interests

The authors declare that they have no competing interests.

## Authors' contributions

KWC: Initiated the study, designed the questionnaire and research strategy, supervised the survey, analyzed the data and wrote up the final manuscript. CCB: Participated in initial research plan and questionnaire design, performed data collection and data entry; conducted literature review and wrote up the introduction. DPF: Participated in initial research plan and questionnaire design, data collection and data entry, helped with literature review and finalizing the manuscript. CDA: Helped with data cleaning, performed statistical analysis, and final manuscript preparation. EW: participated in initial research idea, planning and questionnaire design, supervised the clinic feasibility and data collection, contributed to final manuscript preparation. DG: Initiated the research concept, participated in initial research plan and questionnaire design, performed literature review and clinical planning, contributed to final manuscript preparation.

All authors read and approved the final manuscript.

## Pre-publication history

The pre-publication history for this paper can be accessed here:

http://www.biomedcentral.com/1471-244X/11/90/prepub
